# Three-Color Balancing for Color Constancy Correction

**DOI:** 10.3390/jimaging7100207

**Published:** 2021-10-06

**Authors:** Teruaki Akazawa, Yuma Kinoshita, Sayaka Shiota, Hitoshi Kiya

**Affiliations:** Department of Computer Science, Tokyo Metropolitan University, 6-6 Asahigaoka, Tokyo 191-0065, Japan; akazawa-teruaki@ed.tmu.ac.jp (T.A.); ykinoshita@tmu.ac.jp (Y.K.); sayaka@tmu.ac.jp (S.S.)

**Keywords:** color image processing, color constancy, white balance adjustment, color correction, color distortion

## Abstract

This paper presents a three-color balance adjustment for color constancy correction. White balancing is a typical adjustment for color constancy in an image, but there are still lighting effects on colors other than white. Cheng et al. proposed multi-color balancing to improve the performance of white balancing by mapping multiple target colors into corresponding ground truth colors. However, there are still three problems that have not been discussed: choosing the number of target colors, selecting target colors, and minimizing error which causes computational complexity to increase. In this paper, we first discuss the number of target colors for multi-color balancing. From our observation, when the number of target colors is greater than or equal to three, the best performance of multi-color balancing in each number of target colors is almost the same regardless of the number of target colors, and it is superior to that of white balancing. Moreover, if the number of target colors is three, multi-color balancing can be performed without any error minimization. Accordingly, we propose three-color balancing. In addition, the combination of three target colors is discussed to achieve color constancy correction. In an experiment, the proposed method not only outperforms white balancing but also has almost the same performance as Cheng’s method with 24 target colors.

## 1. Introduction

A change in illumination affects the pixel values of an image taken with an RGB digital camera because the values are determined by spectral information such as the spectra of illumination [1,2,3]. In the human visual system, it is well known that illumination changes (i.e., lighting effects) are reduced, and this ability keeps the entire color perception of a scene constant [4]. In contrast, since cameras do not intrinsically have this ability, white balancing is applied to images [5]. Otherwise, in the field of image segmentation or object recognition, we may suffer from color distortion caused by lighting effects [2,3,6,7,8,9,10].

Applying white balancing requires a two-step procedure: estimating a white region with remaining lighting effects (i.e., a source white point) and mapping the estimated white region into the ground truth white without lighting effects. Many studies have focused on estimating a source white point in images [11,12,13,14,15,16,17,18,19]. However, even when white regions are accurately estimated, colors other than white still include lighting effects. Therefore, various methods for reducing lighting effects on multiple colors have been investigated as in [5,20,21,22,23,24,25,26,27,28,29,30,31]. For example, von Kries’s [20] and Bradford’s [21] chromatic adaptation transforms were proposed to address this problem under the framework of white balancing.

In contrast, Cheng et al. [5] proposed a multi-color balancing for reducing lighting effects on both chromatic and achromatic colors. In Cheng’s method, multiple colors with remaining lighting effects, called “target colors” in this paper, are used for designing a matrix that maps the target colors into ground truth ones. Although Cheng’s method contributes more to improving color constancy correction than white balancing, it still has three problems: choosing the number of target colors, selecting the combination of target colors, and minimizing error which causes the computational complexity to increase.

In this paper, we propose a three-color balance adjustment. This paper has two contributions. The first one is to show that if the combination of three target colors that provides the lowest mean error under three-color balancing is chosen, the three-color balancing will have almost the same performance as Cheng’s method with 24 target colors. The second one is to show that there are multiple appropriate combinations of the three target colors, which can offer a low mean error even under various lighting conditions. From the second, some examples of the combination of three target colors are recommended, which can maintain the performance of three-color balancing under general lighting conditions. As a result, the proposed three-color balancing can more improve color constancy correction than WB. Furthermore, the proposed method gives us a new insight for selecting three target colors in multi-color balancing.

In experiments, three-color balancing is demonstrated to have almost the same performance as Cheng’s method with 24 target colors, and it outperforms conventional white balance adjustments. Additionally, the computational complexity was tested under various numbers of target colors, and the proposed method achieved faster processing than Cheng’s method with error minimization algorithms.

## 2. Related Work

Here, we summarize how illumination change affects colors in a digital image, and white balancing and Cheng’s multi-color balancing are summarized.

### 2.1. Lighting Effects on Digital Images

**Figure 1:**
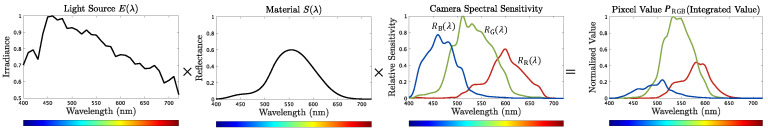
Imaging pipeline of RGB digital camera.

On the basis of the Lambertian model [1], pixel values of an image taken with an RGB digital camera are determined by using three elements: spectra of illumination E(λ), spectral reflectance of objects S(λ), and camera spectral sensitivity $R_{C}$ for color C∈{R,G,B}, where λ spans the visible spectrum in the range of [400,720] (see Figure 1) [2,3]. A pixel value $P_{\mathrm{RGB}}={\left(r_{P},g_{P},b_{P}\right)}^{⊤}$ in the camera RGB color space is given by

(1) $$P_{\mathrm{RGB}}=\int_{400}^{720}\;\;\;\;E(\lambda )S(\lambda )R_{C}\left(\lambda \right)\;d\lambda .$$

Equation (1) means that a change in illumination E(λ) affects the pixel values in an image. In the human visual system, the changes (i.e., lighting effects) are reduced, and the overall color perception is constant regardless of illumination differences, known as chromatic adaptation [4]. To mimic this human ability as a computer vision task, white balancing is typically performed as a color adjustment method.

### 2.2. White Balance Adjustments

By using white balancing, lighting effects on a white region in an image are accurately corrected if the white region under illumination is correctly estimated. Many studies on color constancy have focused on correctly estimating white regions, and a variety of automatic algorithms are available [11,12,13,14,15,17,18].

White balancing is performed by

(2) $$P_{\mathrm{WB}}=M_{\mathrm{WB}}P_{\mathrm{XYZ}}.$$

$M_{\mathrm{WB}}$ in Equation (2) is given as

(3) $$M_{\mathrm{WB}}={M_{A}}^{-1}\left(\begin{array}{ccc} \rho_{D}/\rho_{S} & 0 & 0\\ 0 & \gamma_{D}/\gamma_{S} & 0\\ 0 & 0 & \beta_{D}/\beta_{S} \end{array}\right)M_{A},$$

where $P_{\mathrm{XYZ}}={\left(X_{P},Y_{P},Z_{P}\right)}^{⊤}$ is a pixel value of an input image $I_{\mathrm{XYZ}}$ in the XYZ color space [32], and $P_{\mathrm{WB}}={\left(X_{\mathrm{WB}},Y_{\mathrm{WB}},Z_{\mathrm{WB}}\right)}^{⊤}$ is that of a white-balanced image $I_{\mathrm{WB}}$ [33]. $M_{A}$ with a size of 3 × 3 is decided in accordance with an assumed chromatic adaptation transform [33]. ${\left(\rho_{S},\gamma_{S},\beta_{S}\right)}^{⊤}$ and ${\left(\rho_{D},\gamma_{D},\beta_{D}\right)}^{⊤}$ are calculated from a source white point ${\left(X_{S},Y_{S},Z_{S}\right)}^{⊤}$ in an input image and a ground truth white point ${\left(X_{D},Y_{D},Z_{D}\right)}^{⊤}$ as

(4) $$\left(\;\begin{array}{c} \rho_{S}\\ \gamma_{S}\\ \beta_{S} \end{array}\;\right)=M_{A}\left(\;\begin{array}{c} X_{S}\\ Y_{S}\\ Z_{S} \end{array}\;\right)\;\mathrm{and}\;\left(\;\begin{array}{c} \rho_{D}\\ \gamma_{D}\\ \beta_{D} \end{array}\;\right)=M_{A}\left(\;\begin{array}{c} X_{D}\\ Y_{D}\\ Z_{D} \end{array}\;\right).$$

We call using the 3 × 3 identity matrix as $M_{A}$ “white balancing with XYZ scaling” in this paper. Otherwise, von Kries’s [20] and Bradford’s [21] chromatic adaptation transforms were also proposed for reducing lighting effects on all colors under the framework of white balancing. For example, under the use of von Kries’s model, $M_{A}$ is given as

(5) $$M_{A}=\left(\begin{array}{ccc} 0.4002 & 0.7076 & -0.0808\\ -0.2263 & 1.1653 & 0.0457\\ 0.0000 & -0.0000 & 0.9182 \end{array}\right).$$

### 2.3. Cheng’s Multi-Color Balancing

White balancing is a method that maps a source white point in an image into a ground truth one as in Equation (3). In other words, colors other than white are not considered in this mapping, although the goal of color constancy is to remove lighting effects on all colors. To address this problem, various chromatic adaptation transforms such as those of von Kries [20], Bradford [21], CAT02 [22], and the latest CAM16 [23] have been proposed to reduce lighting effects on all colors under the framework of white balancing.

In addition, Cheng et al. proposed a method [5] for considering both achromatic and chromatic colors to further relax the limitation that white balancing has. In their method, multiple colors are used instead of white. Let $T_{1}^{\prime }={\left(X_{T1}^{\prime },Y_{T1}^{\prime },Z_{T1}^{\prime }\right)}^{⊤}$, $T_{2}^{\prime }={\left(X_{T2}^{\prime },Y_{T2}^{\prime },Z_{T2}^{\prime }\right)}^{⊤}$, $T_{3}^{\prime }={\left(X_{T3}^{\prime },Y_{T3}^{\prime },Z_{T3}^{\prime }\right)}^{⊤}$, ⋯, $T_{n}^{\prime }={\left(X_{Tn}^{\prime },Y_{Tn}^{\prime },Z_{Tn}^{\prime }\right)}^{⊤}$ be *n* colors with remaining lighting effects in the XYZ color space. In this paper, these colors are called “target colors.” Additionally, let $G_{1}^{\prime }={\left(X_{G1}^{\prime },Y_{G1}^{\prime },Z_{G1}^{\prime }\right)}^{⊤}$, $G_{2}^{\prime }={\left(X_{G2}^{\prime },Y_{G2}^{\prime },Z_{G2}^{\prime }\right)}^{⊤}$, $G_{3}^{\prime }={\left(X_{G3}^{\prime },Y_{G3}^{\prime },Z_{G3}^{\prime }\right)}^{⊤}$, ⋯, $G_{n}^{\prime }={\left(X_{Gn}^{\prime },Y_{Gn}^{\prime },Z_{Gn}^{\prime }\right)}^{⊤}$ be *n* ground truth colors corresponding to each target color. To calculate a linear transform matrix, let two matrices $T^{\prime }$ and $G^{\prime }$ be

(6) $$\begin{array}{c} T^{\prime }=\left(\;\;\begin{array}{ccccc} X_{T1}^{\prime } & X_{T2}^{\prime } & X_{T3}^{\prime } & \; & X_{Tn}^{\prime }\\ Y_{T1}^{\prime } & Y_{T2}^{\prime } & Y_{T3}^{\prime } & \cdots & Y_{Tn}^{\prime }\\ Z_{T1}^{\prime } & Z_{T2}^{\prime } & Z_{T3}^{\prime } & \; & Z_{Tn}^{\prime } \end{array}\;\;\;\right)\mathrm{and}\;G^{\prime }=\left(\;\;\begin{array}{ccccc} X_{G1}^{\prime } & X_{G2}^{\prime } & X_{G3}^{\prime } & \; & X_{Gn}^{\prime }\\ Y_{G1}^{\prime } & Y_{G2}^{\prime } & Y_{G3}^{\prime } & \cdots & Y_{Gn}^{\prime }\\ Z_{G1}^{\prime } & Z_{G2}^{\prime } & Z_{G3}^{\prime } & \; & Z_{Gn}^{\prime } \end{array}\;\;\;\right), \end{array}$$

respectively. In Cheng’s method, *n* was set to 24 (i.e., all patches in a color rendition chart) [5]. If *n* is greater than three, $T^{\prime }$ and $G^{\prime }$ will be a singular matrix, and the inverse matrix of $T^{\prime }$ is not uniquely determined. Hence, the Moore–Penrose pseudoinverse [5,34] is used, and the optimal linear transform matrix $M^{+}$ is given as

(7) $$M^{+}=G^{\prime }{T^{\prime }}^{⊤}{\left(T^{\prime }{T^{\prime }}^{⊤}\right)}^{-1}.$$

However, as noted by Funt et al. [35], because the illumination across the target colors is generally not uniform, calculating $M^{+}$ only with Equation (7) is insufficient to perform calibration for such circumstances. Hence, with $M^{+}$ calculated in Equation (7) as the input, the method in [35] is also used to minimize the sum of error:

(8) $$M^{†}=\underset{M^{+}}{\mathrm{argmin}}\sum_{i=1}^{n}\cos^{-1}\left(\frac{M^{+}T_{i}^{\prime }\cdot G_{i}^{\prime }}{\|M^{+}T_{i}^{\prime }\left\|\right\|G_{i}^{\prime }\|}\right).$$

As well as white balancing, a pixel value corrected by Cheng’s method $P_{\mathrm{CNG}}$ is given as

(9) $$P_{\mathrm{CNG}}=M^{†}P_{\mathrm{XYZ}},$$

where $M^{†}$ is calculated as in Equations (6)–(8).

However, this method still has three problems that have not been discussed:

(i) How the number of target colors is decided.
(ii) How the combination of *n* target colors is selected.
(iii) How the computational complexity of Cheng’s method is reduced.

Additionally, as for (iii), if the number of target colors is increased, the computational complexity of Cheng’s method will be increased due to the use of Equation (8).

## 3. Proposed Method

In this section, we investigate the relationship between the number of target colors and the performance of Cheng’s multi-color balancing. From the investigation, we point out that if the combination of three target colors that offers the lowest mean error in three-color balancing is chosen, the three-color balancing will have almost the same performance as Cheng’s method with 24 target colors. Accordingly, we propose a three-color balance adjustment that maps three target colors into corresponding ground truth colors without minimizing error. Additionally, the selection of three target colors is discussed, and we recommend some example combinations of three target colors, which can be used under general illumination. Finally, the procedure of the proposed method is summarized.

### 3.1. Number of Target Colors

In this section, we argue the relationship between the number of target colors and the performance of color constancy correction.

**Figure 2:**
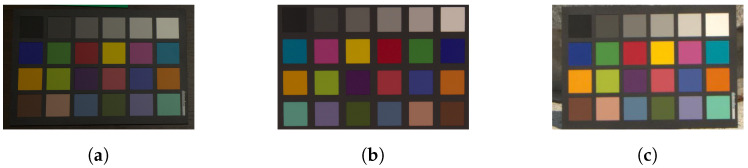
Color rendition charts under different light sources. (**a**) Ground truth color rendition chart, (**b**) color rendition chart under artificial light source, and (**c**) color rendition chart under daylight.

White balancing with XYZ scaling and Cheng’s multi-color balancing were applied to the images in Figure 2b,c, respectively, where *n* was set to 1 for white balancing, and it was set to 3, 4, 5, and 24 for Cheng’s method. Additionally, corresponding ground truth colors were selected from Figure 2a.

**Figure 3:**
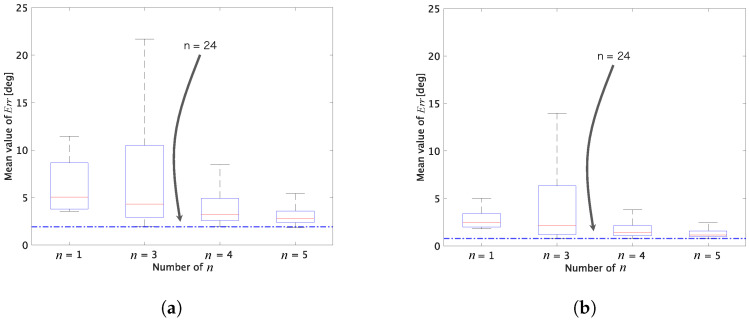
Box plots of mean values of reproduction errors for 24 patches. (**a**) Image in Figure 2b. (**b**) Image in Figure 2c. Boxes span from the first to third quartile, referred to as $Q_{1}$ and $Q_{3}$. Whiskers show maximum and minimum values in range of $\left[Q_{1}-1.5\left(Q_{3}-Q_{1}\right),Q_{3}+1.5\left(Q_{3}-Q_{1}\right)\right]$. The red band inside boxes indicates the median.

Figure 3 shows box plots of experimental results under various conditions. In each adjustment, all combinations of *n* target colors were chosen from 24 patches in the color rendition chart. Therefore, the combination number of *n* target colors was ${}_{24}C_{n}$. For example, when n=1, there are 24 combinations of target colors.

For every combination of target colors, the performance of each adjustment was evaluated by using the mean value of reproduction angular errors for the 24 patches. The reproduction angular error is given by

(10) $$Err=\frac{180}{\pi }\;\cos^{-1}\left(\frac{P\cdot Q}{∥P∥∥Q∥}\right)\;\;\;\left[\deg\right]\;,$$

where P is a mean-pixel value of an adjusted patch, and Q is that of the corresponding ground truth one [36]. In this experiment, P corresponds to an adjusted patch in Figure 2b,c, and Q corresponds to a patch in Figure 2a. From the figure, two properties are summarized as follows.

(i) Cheng’s method had a lower minimum mean error than white balancing.
(ii) When n≥3, Cheng’s method had almost the same minimum mean error as that of n=24.

Moreover, when n=3 is chosen, $M^{+}$ is reduced to $M^{†}$ if $T^{\prime }$ and $G^{\prime }$ have full rank, so Equation (8) is not required. Accordingly, in this paper, we propose selecting n=3.

### 3.2. Proposed Three-Color Balancing

**Figure 4:**
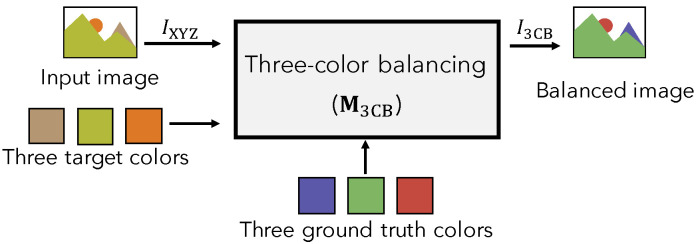
Overview of proposed method.

Figure 4 shows an overview of the proposed three-color balancing. In a manner like white balancing, three-color balanced pixel $P_{3\mathrm{CB}}$ is given as

(11) $$P_{3\mathrm{CB}}=M_{3\mathrm{CB}}P_{\mathrm{XYZ}}.$$

Let $T_{1}={\left(X_{T1},Y_{T1},Z_{T1}\right)}^{⊤}$, $T_{2}={\left(X_{T2},Y_{T2},Z_{T2}\right)}^{⊤}$, and $T_{3}={\left(X_{T3},Y_{T3},Z_{T3}\right)}^{⊤}$ be three target colors in the XYZ color space. Note that the location of each target color is known; or target colors can be estimated by using a color estimation algorithm although color estimation algorithms do not have enough performance data yet for estimating various colors [37,38,39,40]. Additionally, let $G_{1}={\left(X_{G1},Y_{G1},Z_{G1}\right)}^{⊤}$, $G_{2}={\left(X_{G2},Y_{G2},Z_{G2}\right)}^{⊤}$, and $G_{3}={\left(X_{G3},Y_{G3},Z_{G3}\right)}^{⊤}$ be corresponding ground truth colors, respectively. Then, $M_{3\mathrm{CB}}$ satisfies

(12) $$G=M_{3\mathrm{CB}}T,$$

where

(13) $$\begin{array}{c} T=\left(\begin{array}{ccc} X_{T1} & X_{T2} & X_{T3}\\ Y_{T1} & Y_{T2} & Y_{T3}\\ Z_{T1} & Z_{T2} & Z_{T3} \end{array}\right)\mathrm{and}\;G=\left(\;\;\begin{array}{ccc} X_{G1} & X_{G2} & X_{G3}\\ Y_{G1} & Y_{G2} & Y_{G3}\\ Z_{G1} & Z_{G2} & Z_{G3} \end{array}\right). \end{array}$$

When both T and G have full rank, $M_{3\mathrm{CB}}$ is designed by

(14) $$M_{3\mathrm{CB}}=GT^{-1}.$$

By applying $M_{3\mathrm{CB}}$ in Equation (14) to every pixel value in input image $I_{\mathrm{XYZ}}$, balanced image $I_{3\mathrm{CB}}$ is obtained, where target colors in $I_{\mathrm{XYZ}}$ are mapped into ground truth ones.

The proposed method is considered as a special case of Cheng’s multi-color balancing. If the number of target colors is three and both T and G have full rank, $T^{-1}$ is uniquely determined, and $M_{3\mathrm{CB}}$ can be designed without any error minimization algorithms.

### 3.3. Selection of Three Target Colors

Under one light source, the optimal combination of the three target colors has to be selected by testing all of the conceivable combinations, as discussed in Section 3.1. However, because illumination continuously changes in real situations, it is difficult to repeat performing the selection for every light source. Accordingly, in this section, appropriate combinations of three target colors, which offer a low mean reproduction error are recommended by experimentally testing all combinations of three target colors for 500 images taken under various light sources.

**Figure 5:**
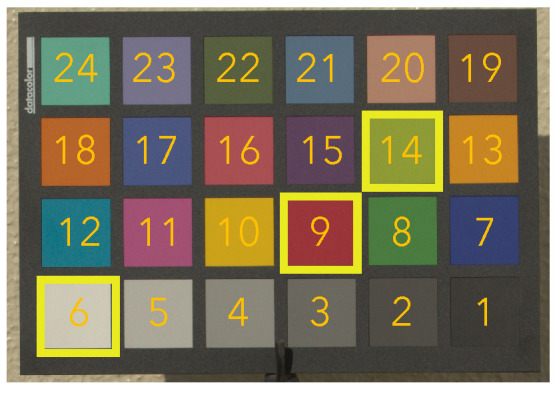
Example of the selected three colors, which are highlighted by yellow squares. Each patch is numbered from 1 to 24, and patches indexed by 6 (white), 9 (red), and 14 (yellow green) were selected.

We used the ColorChecker dataset prepared by Hemrit et al. [41], in which pixel values of the 24 patches in a color rendition chart were recorded under 551 illumination conditions. Additionally, the ground truth colors selected in Figure 2a were used for this discussion. By using 500 images in the dataset, all combinations of three colors were tested, and the performance of the proposed method with each combination was evaluated in terms of the mean reproduction error for the 24 patches. From the experiment, a combination of three colors (index 6, 9, and 14 in Figure 5) was selected at which the proposed method had the minimum value among 500 mean Err values.

Additionally, we used the other 51 images from the dataset to evaluate the effectiveness of the combination of three target colors. In Table 1, three-color balancing was compared with Cheng’s method and white balancing with XYZ scaling, referred to as 3CB (6, 9, 14), Cheng (1–24), and WB (XYZ), respectively. The selected combination shown in Figure 5 was used for three-color balancing, and all of the 24 target colors were chosen for Cheng’s method. For white balancing, the white patch (index 6) was selected.

From Table 1, 3CB (6, 9, 14) had almost the same performance as Cheng (1–24). In addition, 3CB (6, 9, 14) and Cheng (1–24) outperformed WB (XYZ) for almost all chromatic colors (index 7–24). In other words, WB (XYZ) did not reduce lighting effects on colors other than achromatic colors (index 1–6).

**Figure 6:**
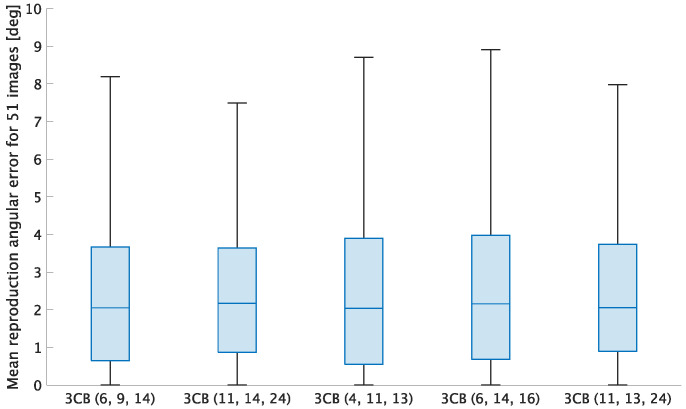
Box plot of three-color balancing with five combinations of target colors. Boxes span from the first to third quartile, referred to as $Q_{1}$ and $Q_{3}$. Whiskers show maximum and minimum values in range of $\left[Q_{1}-1.5\left(Q_{3}-Q_{1}\right),Q_{3}+1.5\left(Q_{3}-Q_{1}\right)\right]$. The band inside boxes indicates the median.

Figure 6 shows box plots of three-color balancing with four more combinations of three target colors in addition to 3CB (6, 9, 14), where 3CB (6, 9, 14), 3CB (11, 14, 24), 3CB (4, 11, 13), 3CB (6, 14, 16), and 3CB (11, 13, 24) had the first, second, third, fourth, and fifth smallest mean reproduction error, respectively.

From the figure, the five combinations of three target colors had almost the same result. This means that the effectiveness of three-color balancing can be maintained even when (6, 9, 14) is replaced with other combinations. Therefore, for example, we recommend (6, 9, 14) and (11, 14, 24) from Figure 6 as the top two combinations of target colors, which can maintain high performance under general illumination.

### 3.4. Procedure of Three-Color Balancing

When we assume that three colors selected from 24 colors in Figure 2a are used as ground truth colors, the procedure of the three-color balancing is given as below.

(i) Select three ground truth colors from Figure 2a. Let them $G_{1}$, $G_{2}$, and $G_{3}$, respectively.
(ii) Prepare three objects in a camera frame, in which each object color corresponds to one of the ground truth colors. Compute three target colors $T_{1}$, $T_{2}$, and $T_{3}$ from the region of each object, respectively.
(iii) Apply three-color balancing by using $T_{1}$, $T_{2}$, and $T_{3}$ and $G_{1}$, $G_{2}$, and $G_{3}$, following Section 3.2.

Using (6, 9, 14) or (11, 14, 24) as a combination is recommended in step (i), as discussed in Section 3.3. Additionally, if the ground truth colors other than those from Figure 2a are used, the selection of target colors discussed in Section 3.3 should be carried out by using the newly determined ground truth colors.

## 4. Experiment

We conducted experiments to confirm the effectiveness of the proposed method.

### 4.1. Evaluation of Reducing Lighting Effects

**Figure 7:**
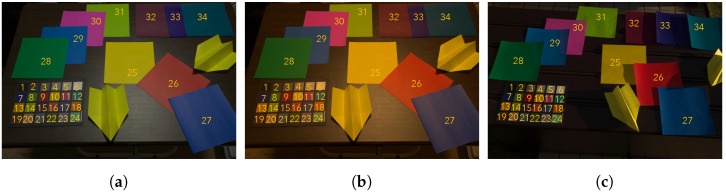
Images taken under three different light sources. (**a**) Ground truth image, (**b**) image taken under warm white light, and (**c**) image taken under daylight.

In this experiment, the effectiveness of three-color balancing with the recommended combinations was demonstrated by using various colors and lighting conditions, where these experimental conditions were different from those in Section 3.3. Figure 7 shows images taken under three different light sources, where 10 more color regions were added to the color rendition chart in Figure 2, and the images included 34 color regions numbered from 1 to 34. Additionally, the lighting condition in Figure 7a is the same as that of Figure 2a, which means that the color regions numbered from 1 to 24 in Figure 7a correspond to those of Figure 2a.

**Figure 8:**
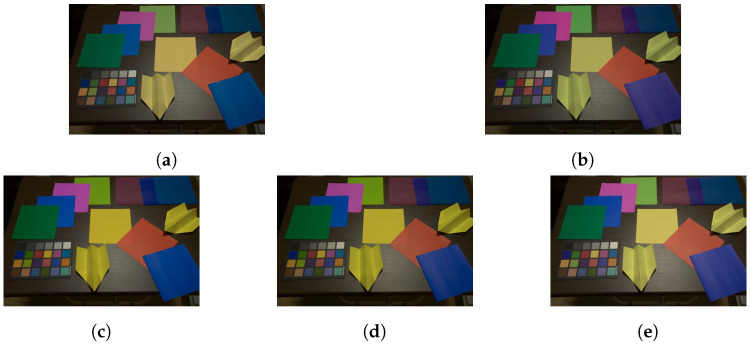
Images adjusted from Figure 7b. (**a**) WB (XYZ), (**b**) WB (von Kries), (**c**) 3CB (6, 9, 14), (**d**) 3CB (11, 14, 24), and (**e**) Cheng (1–24).

**Figure 9:**
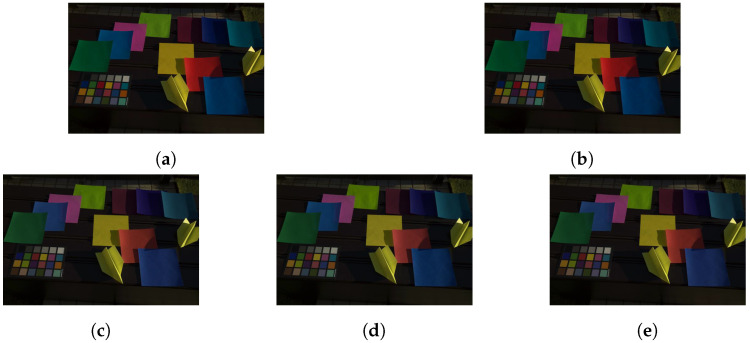
Images adjusted from Figure 7c. (**a**) WB (XYZ), (**b**) WB (von Kries), (**c**) 3CB (6, 9, 14), (**d**) 3CB (11, 14, 24), and (**e**) Cheng (1–24).

Representative RGB values were computed as the mean-pixel value of a color region in Figure 7. Figure 8 and Figure 9 show images adjusted by using various methods.

The proposed method was applied to the input images, and it was compared with white balancing and Cheng’s method (Cheng (1–24)), where XYZ scaling (WB (XYZ)) and von Kries’s model (WB (von Kries)) were applied as white balance adjustments. Target colors were selected from the color rendition chart indexed from 1 to 24 in Figure 7b,c. For white balancing, the white region (index 6) was chosen. Additionally, for Cheng’s method, all of the 24 color regions were used as target colors. For the proposed method, two combinations of the three target colors determined in Section 3.3 were selected: (6, 9, 14) and (11, 14, 24), referred as to 3CB (6, 9, 14) and 3CB (11, 14, 24), respectively.

In Table 2 and Table 3, the reproduction angular errors for Equation (10) are shown to objectively compare the corrected images. From the tables, the proposed method had almost the same performance as Cheng’s method with 24 target colors in terms of the mean value and standard variation. Additionally, the proposed method and Cheng’s method outperformed conventional white balance adjustments, although the mean value of 3CB (6, 9, 14) had a higher error than that of WB (von Kries) in Table 2. Therefore, the effectiveness of the proposed method for color constancy correction was confirmed.

### 4.2. Evaluation of Computational Complexity

In Cheng’s method, all of the 24 patches in a color rendition chart are used as target colors. Additionally, when n≥4, the computational complexity of Cheng’s method will increase due to the use of Equation (8). In contrast, that of three-color balancing is low because Equation (8) is not required.

To evaluate the complexity, we implemented white balancing with XYZ scaling, three-color balancing, and Cheng’s method on a computer with a 3.6 GHz processor and a main memory of 16 Gbytes (see Table 4).

**Figure 10:**
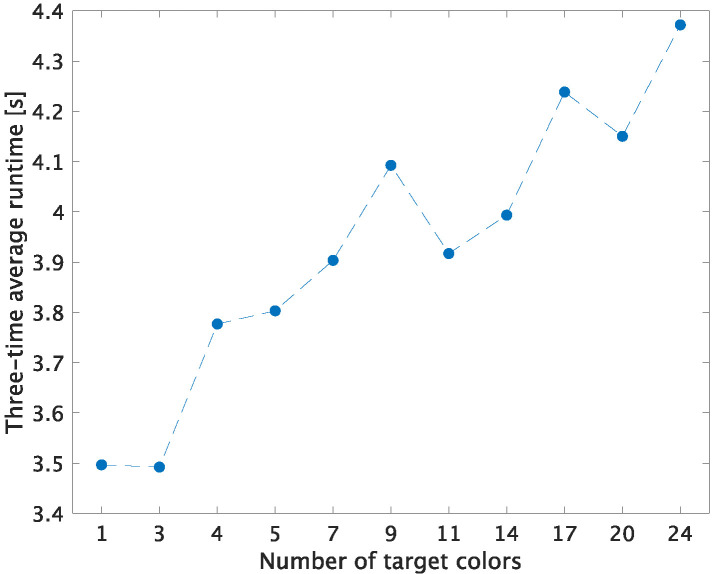
Three-time average runtimes for applying white balancing, three-color balancing, and Cheng’s method to 51 images. Among number of target colors, 1, 3, and over 4 indicate white balancing, three-color balancing, and Cheng’s method, respectively.

Note that the runtime of the equations shown in Section 2.2, Section 2.3, and Section 3.2 was only measured for white balancing, Cheng’s method, and three-color balancing, respectively. In other words, for example, image loading runtimes were excluded from the measurement. Figure 10 shows the runtime result in the case that each method was applied to the 51 test images in Section 3.3.

As shown in Figure 10, while the proposed three-color balancing and white balancing had almost the same average runtime, Cheng’s method required longer runtimes to minimize error. Moreover, in Cheng’s method, if the number of target colors increases, it will result in a long runtime.

## 5. Discussion on the Rank of **T and G**

**Figure 11:**
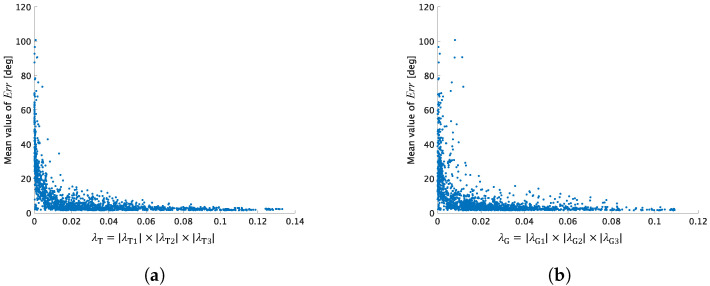
Product of three eigenvalues and mean value of reproduction error (Err) for 24 patches. (**a**) Eigenvalues were calculated from T designed under ${}_{24}C_{3}$ combinations of three target colors. (**b**) Eigenvalues were calculated from G designed under corresponding ground truth colors.

In the proposed three-color balancing, when T and G have full rank, $M_{3\mathrm{CB}}$ can be designed as in Equation (14). If T and G violate the rank constraints, their eigenvalues will include an almost zero value due to rank deficiency [42]. Figure 11 shows the relationship between three eigenvalues of T or G and the performance of three-color balancing under various combinations of three colors. In the figure, for visualization, the product of the three eigenvalues was calculated for the horizontal axis:

(15) $$\lambda_{T}=|\lambda_{T1}\left|\times \right|\lambda_{T2}\left|\times \right|\lambda_{T3}|\;\;\;\mathrm{and}\;\;\;\lambda_{G}=|\lambda_{G1}\left|\times \right|\lambda_{G2}\left|\times \right|\lambda_{G3}|,$$

where $\lambda_{T1},\;\lambda_{T2}$, and $\lambda_{T3}$ denote eigenvalues calculated from T, and $\lambda_{G1},\;\lambda_{G2}$, and $\lambda_{G3}$ denote those from G. $\lambda_{T}$ was calculated by using various combinations of three target colors selected from Figure 2a, and $\lambda_{G}$ was calculated by using corresponding ground truth colors from Figure 2b.

In a manner like Figure 3, the performance of three-color balancing for every combination was evaluated in terms of the mean reproduction values for the 24 patches. From the figure, we confirmed that if one eigenvalue is nearly zero, i.e., the product of the eigenvalues is nearly zero, three-color balancing results in a high mean Err value. This means that the rank deficiency caused by the combination of linearly dependent color vectors significantly affects the overall color correction performance. Thus, the following results were verified:

(i) If the product of eigenvalues is small, the mean value of reproduction errors will be high.
(ii) There are many combinations of three target colors at which three-color balancing results in a high error.
(iii) Analysis based on eigenvalues enables us to select three target colors without a large dataset as in Section 3.3.

## 6. Conclusions

In this paper, we proposed a three-color balance adjustment for color constancy. While conventional white balancing cannot perfectly adjust colors other than white, multi-color balancing including three-color balancing improves color constancy correction by using multiple colors. Additionally, this paper presented the choice of the number of *n* target colors and the selection of three target colors for the proposed three-color balancing. When the number of target colors is over three, the best performance of color constancy correction is almost the same regardless of *n*. Additionally, for three-color balancing, a combination of three target colors that enables us to achieve a lower reproduction error was determined with a large dataset. Moreover, no algorithms for minimizing error are required to use the proposed method, and this contributes to reducing computational complexity. Experimental results indicated that the proposed three-color balancing did not only outperform conventional white balancing but also had almost the same performance as multi-color balancing with 24 target colors. 

## Figures and Tables

**Table 1:** Mean error (Mean) and standard variation (Std) of Err values for every patch (deg). Each color index corresponds to those of Figure 5.

Color Index	3CB (6, 9, 14)		Cheng (1–24)		WB (XYZ)	
	Mean	Std	Mean	Std	Mean	Std
1	2.5141	2.4884	2.1365	2.0672	1.5416	1.4212
2	1.3788	1.0894	1.2562	0.7173	0.9078	0.6043
3	0.7882	0.6443	0.4805	0.3028	0.5971	0.4157
4	0.4329	0.2943	0.7205	0.6967	0.3046	0.1843
5	0.3216	0.2180	0.7507	0.7153	0.2194	0.1317
6	0.0000	0.0000	0.7220	0.8707	0.0000	0.0000
7	10.8271	2.5743	9.8906	2.9990	17.4603	2.4351
8	2.5571	1.4876	2.4314	1.6288	10.1905	1.8228
9	0.0000	0.0000	1.3855	1.3684	7.6581	2.1020
10	2.9289	0.9281	2.4285	1.3505	9.6169	1.3484
11	1.6106	1.5112	0.9028	1.0507	4.9940	1.2577
12	3.6375	1.6300	2.7855	1.5475	7.9601	2.1248
13	3.0252	0.9058	2.4637	0.9592	9.5734	1.2001
14	0.0000	0.0000	0.7588	0.5271	11.3374	1.5411
15	6.2178	1.4381	5.4511	1.3088	10.3233	1.5883
16	1.6448	0.8870	1.7227	0.6843	5.6875	1.2939
17	4.0888	1.3687	3.5478	1.4519	10.2029	1.4913
18	3.1200	0.9860	2.5865	1.1576	7.4177	1.0589
19	2.0731	0.6608	2.0568	0.9368	5.6920	1.2514
20	2.9204	0.8567	3.4227	0.9647	5.4026	0.7180
21	4.1982	1.6043	3.4816	1.2573	8.1818	0.9399
22	4.2825	2.0237	3.8826	1.8114	3.6448	1.1974
23	2.7941	1.2718	2.0937	0.8969	6.9380	0.8475
24	1.5294	1.1186	1.3261	0.8383	3.1651	0.4340
Total Average	2.6205	1.0828	2.4452	1.1712	6.2090	1.1421

**Table 2:** Reproduction angular error (Err) for every object (deg) in Figure 7b. Color indices correspond to those in Figure 7. Indices inside () indicate target colors used in each adjustment.

Color	Pre-Correction	WB (XYZ)	WB (von Kries)	3CB	3CB	Cheng
Index		(6)	(6)	(6, 9, 14)	(11, 14, 24)	(1–24)
1	21.3566	0.6031	0.5168	0.7235	1.9737	0.3500
2	21.0226	0.6441	0.5657	0.8669	1.6121	0.0000
3	21.1741	0.6068	0.5224	0.8709	1.6202	0.0870
4	21.1792	0.4491	0.3957	0.6052	1.8573	0.2267
5	21.3249	0.1997	0.1569	0.3129	2.1111	0.4601
6	21.3115	0.0000	0.0000	0.0000	2.3715	0.6490
7	18.7388	5.5467	1.4326	6.1466	3.9072	1.7944
8	10.4722	3.3503	5.1517	7.6731	8.2366	0.7390
9	10.3560	0.8337	0.6933	0.0000	1.6542	2.7531
10	5.8589	9.1107	9.4896	2.5561	3.1016	8.3993
11	25.5364	2.6138	3.7142	1.9130	0.0000	1.1718
12	22.3124	7.7740	6.0355	10.8480	7.3155	5.9673
13	4.9612	8.1911	8.6001	3.0538	4.0687	8.4872
14	6.4692	8.7257	9.2496	0.0000	0.0000	6.6441
15	29.2100	1.5731	2.2792	4.1710	1.1836	0.6999
16	15.3806	2.4103	2.3149	1.8067	1.6961	1.1918
17	25.9012	4.6039	1.6651	7.3691	4.3680	2.2146
18	5.7546	4.3316	4.7537	0.5211	1.9654	5.5090
19	15.1058	2.3229	1.9819	5.3252	5.2026	1.9077
20	16.3699	1.5752	1.0558	3.2633	3.6401	0.8342
21	25.3728	4.2875	2.5926	7.4420	3.8807	2.6824
22	14.5741	0.8098	1.6973	7.7538	8.2749	2.4076
23	26.6287	2.4779	1.3060	6.0191	2.7302	1.5043
24	19.9906	3.9184	3.9094	3.4344	0.0000	2.1738
25	6.2529	9.0649	9.4082	2.0700	2.4467	8.0168
26	11.9770	3.1357	2.9722	4.0141	2.5103	1.0617
27	19.8988	7.5012	4.1785	9.5476	6.9960	4.9360
28	10.9416	9.0790	10.3906	3.7961	3.0529	4.8839
29	17.2530	7.2625	3.8732	8.6359	6.3031	4.4513
30	25.1127	2.6489	0.9564	6.6488	4.6798	2.7824
31	6.2739	10.9320	11.4670	0.9847	0.6948	8.0793
32	21.7234	2.7743	3.0355	0.1029	1.4113	1.4194
33	25.9394	3.8417	2.1041	4.7243	2.1929	0.5683
34	16.6815	11.8302	9.5212	14.5191	11.5521	9.7971
Mean	17.3064	4.2656	3.7643	4.0506	3.3709	3.0838
Std	7.1416	3.4288	3.4384	3.6313	2.6588	2.9065

**Table 3:** Reproduction angular error (Err) for every object (deg) in Figure 7c. Color indices correspond to those in Figure 7. Indices inside () indicate target colors used in each adjustment.

Color	Pre-Correction	WB (XYZ)	WB (von Kries)	3CB	3CB	Cheng
Index		(6)	(6)	(6, 9, 14)	(11, 14, 24)	(1–24)
1	0.8814	6.3543	6.2129	5.5332	5.7248	5.6021
2	4.4683	0.7990	0.7815	0.7063	1.0852	0.6697
3	5.1001	0.2931	0.2865	0.2713	0.6299	0.1274
4	5.1878	0.2623	0.2638	0.2576	0.5557	0.1277
5	5.2214	0.2175	0.2176	0.2181	0.4927	0.1185
6	5.1013	0.0000	0.0000	0.0000	0.3959	0.1461
7	2.1857	3.2256	1.5804	0.2953	1.1222	0.1872
8	2.4892	1.7274	2.8237	1.2644	1.9037	1.1336
9	8.6557	6.8164	6.8918	0.0000	2.0909	0.6231
10	2.9023	1.9264	1.3637	2.1141	2.6862	1.9947
11	10.1624	4.8819	5.3403	1.0879	0.0000	1.4796
12	3.2927	3.9580	3.3680	0.3680	0.4023	0.5244
13	2.9283	1.8933	1.4585	3.0098	3.9102	2.7644
14	2.2600	0.6336	0.3951	0.0000	0.0000	0.0139
15	6.0898	1.6595	2.3525	1.7127	1.9511	1.8289
16	7.6931	4.4924	4.5682	0.8175	2.1341	0.3307
17	1.7278	4.0290	2.9039	1.6969	2.2152	1.7242
18	5.8159	4.4339	4.2020	0.4470	1.6816	0.0214
19	7.3147	4.1785	4.0182	1.1471	0.3458	1.3105
20	4.8872	1.2518	1.1024	1.1486	1.7719	0.9867
21	4.8807	1.8282	1.2599	0.6607	0.8241	0.5465
22	6.3007	3.4931	3.6341	3.8759	4.0238	3.8070
23	5.8905	0.6857	0.5215	0.7623	0.6639	0.7583
24	3.1728	2.4708	2.5870	0.5151	0.0000	0.6320
25	3.0191	1.8642	1.1719	1.4724	1.9303	1.4009
26	12.4886	11.0326	11.0700	4.3092	2.7438	4.9001
27	2.4211	6.1262	4.9035	3.3014	3.7525	3.3507
28	0.3467	4.6077	4.9793	2.9638	2.2566	2.9399
29	4.4097	1.9139	1.1850	2.4622	2.0271	2.4150
30	9.8741	4.2972	4.6985	2.3209	2.5076	2.1553
31	0.2509	1.6257	2.6189	0.4321	0.3443	0.5715
32	12.6669	8.5415	8.8171	3.2976	2.2430	3.8179
33	1.1494	4.0987	2.5416	1.7988	2.5675	1.7067
34	3.2594	2.4334	1.7380	1.7225	1.0925	1.6765
Mean	4.8381	3.1780	2.9958	1.5291	1.7081	1.5410
Std	3.1739	2.5181	2.5712	1.3827	1.3281	1.4438

**Table 4:** Machine specs used for evaluating runtime.

Processor	Intel Core i7-7700 3.60 GHz
Memory	16 GB
OS	Windows 10 Home
Software	MATLAB R2020a

## Data Availability

The data presented in this study are available on request from the corresponding author.
